# Roll Angle Measurement for a Spinning Vehicle Based on GPS Signals Received by a Single-Patch Antenna

**DOI:** 10.3390/s18103479

**Published:** 2018-10-16

**Authors:** Zilong Deng, Qiang Shen, Zhaowei Deng

**Affiliations:** School of Mechatronical Engineering, Beijing Institute of Technology, Beijing 100081, China; 2120170199@bit.edu.cn (Z.D.); 2220170072@bit.edu.cn (Z.D.)

**Keywords:** GPS based roll angle and rotational speed measurement, single-patch antenna, spinning vehicle

## Abstract

Roll angle measurement is an essential technology in the trajectory correction projectiles. In this paper, an algorithm to detect the roll angle and rotational speed of a spinning vehicle is studied by using a GPS (Global Positioning System) receiver with a single side-mounted antenna. A Frequency-Locked Loop (FLL) assisted Phase-Locked Loop (PLL) is designed to obtain the attitude information from GPS signals, and the optimal parameters of this system are discussed when different rotational speeds are considered. The error estimation of this method and signal-to-noise ratio analysis of GPS signals are also studied. Finally, experiments on the rotary table were carried out to verify the proposed method. The experimental results showed that the proposed algorithm can detect the roll angle in a precision of within 5 degrees.

## 1. Introduction

For some guided spinning projectiles, it is essential to measure the roll angle and rotational speed in real time when it is in the air, because the control surfaces must be correctly actuated to maintain a right trajectory to the target. There are various sensors to detect the roll angle of a spinning projectile in which magnetic and inertial sensors are widely applied, but with some defects. The French-German Research Institute of Saint-Louis have attempted to correct the path of an air-defense projectile using two embedded magnetometers. However, the distortions caused by a ferromagnetic body and changeable geomagnetic environment cannot be modeled and figured out easily to improve the precision of angular information [1]. Park and Kim [2] presented a roll angle estimation algorithm using pitch and yaw rate gyroscopes as well as the Extended Kalman Filter (EKF), which works only under low spinning speed. Moreover, it is difficult to estimate rotational information with a low-cost MEMS (Micro-electromechanical Systems) gyroscope which has a low-dynamic range, because ordinary inertial sensors cannot work properly under the harsh conditions of gun-lunching projectiles [3].

In addition to magnetometers and inertial sensors, roll information can also be determined by GPS [4,5,6]. There are several kinds of GPS-based attitude determining methods, such as the carrier phase-based [7,8], amplitude-based [9,10], and SNR (Signal-to-noise ratio)-based methods, for coarse attitude determination [11]. GPS receivers on guided projectiles normally provide the guidance computer with information of position and velocity. However, it is difficult to obtain position and velocity using receivers with a side-mounted single antenna. Shen and Li [12] proposed a method to track the discontinuous signal received by a single-patch antenna mounted on the side of a spinning vehicle and got the real-time position of it, which enables a GPS receiver to locate and get both the roll angle and roll rate of a spinning vehicle, at the same time. Therefore, the functions of location and roll angle detection can be realized by the study of GPS-based roll angle measurement with only a receiver, which is of great significance in practice for the miniaturization and low cost of projectiles.

Some institutions and corporations have carried out in-depth research, such as Interstate Electronics Corporation [13], May Flower [14], and Honeywell [15]. There is a technology called Advanced Spinning Vehicle Navigation (ASVN), developed by Rockwell Collins, which utilizes the amplitude and phase modulation of GPS signals caused by vehicle rotation to obtain the roll angle of a spinning vehicle. The received signals are used to detect a projectile’s spinning parameters by adding a rotation-demodulating module prior to the correlation of the GPS signals [16,17,18]. Alexander and Redhead [13] proposed a method for determining the rotational attitude of a spinning vehicle by the value of one-millisecond accumulation results in the tracking process of the receiver. However, all of these works lack profound analysis in theory, and do not give a set of specific parameters to be realized, as well as not mentioning the parameter optimization method.

Based on the existing research, we explain and analyze the mechanism of drawing the angular information from the satellite signals received by a single side-mounted antenna, seeking a way to improve the precision of roll angle and rotational speed by means of the signal communication processing method, namely, the phase-locked loops.

Firstly, the formation mechanism and extraction method of roll modulation signals, which are amplitude modulated signals encoded with rotation information, are analyzed. Then the roll angle detection method based on Frequency-Locked Loop (FLL)-assisted Phase-Locked Loop (PLL) is described and the specific parameters are studied and optimized. Lastly, experiments are carried out to verify the proposed method. The experimental results show that the proposed method can work properly with acceptable precision.

## 2. Analysis of Modulation Process

Before obtaining the roll angle from GPS signals, it is necessary to clarify the modulation process during antenna rotation, in order to figure out the true roll angle from the phases of roll modulation signals.

### 2.1. The Models of Antenna and Spinning Vehicle

**Figure 1:**
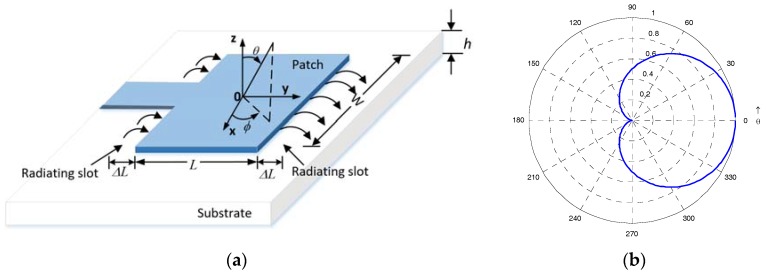
Schematic diagram (**a**) and radiation pattern (**b**) of the rectangular microstrip antenna.

The microstrip antenna has been extensively applied to GNSS (Global Navigation Satellite System) receivers for its low cost and low profile [19]. In this paper, a microstrip single-patch antenna is taken as an example to analyze its radiation pattern and the effect of rotation on the amplitude of received signals. A local spherical coordinate system is set up at the center of the microstrip antenna, as shown in Figure 1a,b. The figure shows a typical normalized radiation pattern for the microstrip antenna.

According to transmission line theory [20], the normalized far-field radiation pattern of a microstrip antenna can be expressed as:

(1) $$E_{\theta }=\cos\phi \frac{\sin\left(\frac{k_{0}h}{2}\sin\theta \cos\phi \right)}{\frac{k_{0}h}{2}\sin\theta \cos\phi }\cdot \frac{\sin\left(\frac{k_{0}W}{2}\sin\theta \sin\phi \right)}{\frac{k_{0}W}{2}\sin\theta \sin\phi }\cos\left(\frac{k_{0}L_{e}}{2}\sin\theta \cos\phi \right)\;$$

(2) $$E_{\phi }=\cos\theta \sin\phi \frac{\sin\left(\frac{k_{0}h}{2}\sin\theta \cos\phi \right)}{\frac{k_{0}h}{2}\sin\theta \cos\phi }\cdot \frac{\sin\left(\frac{k_{0}W}{2}\sin\theta \sin\phi \right)}{\frac{k_{0}W}{2}\sin\theta \sin\phi }\cos\left(\frac{k_{0}L_{e}}{2}\sin\theta \sin\phi \right)\;$$

where *k*_0_ is the wave number, *k*_0_ *=* 2π*/λ*, *λ* is the length of the wave, *L_e_ = L +* 2Δ*L* is the effective length of the antenna, *L* is the length of the antenna, Δ*L* is the length of radiating slots, *W* is the width, *h* is the thickness of the antenna.

Therefore, the radiation pattern of E surface (*φ* = 0) is given by:

(3) $$E_{\theta }=\sin\left(\frac{k_{0}h}{2}\sin\theta \right)\cos\left(\frac{k_{0}L_{e}}{2}\sin\theta \right)/\frac{k_{0}h}{2}\sin\theta \;$$

The radiation pattern of H surface (*φ* = π/2) is given by:

(4) $$E_{\phi }(\theta )=\cos\theta \sin\left(\frac{k_{0}W}{2}\sin\theta \right)/\frac{k_{0}W}{2}\sin\theta \;$$

The relationship between the radiation pattern and the power of the receiver signals *P_R_*(*θ*,*φ*) can be expressed as follows:

(5) $$P_{R}\left(\theta ,\phi \right)={\left(\frac{\lambda }{4\pi d}\right)}^{2}P_{T}G_{R}G_{T}={\left(\frac{\lambda }{4\pi d}\right)}^{2}P_{T}\eta_{A}D(\theta ,\phi )G_{T}\;$$

where *λ* is the wavelength of signal, *d* is the transmission distance, *P_T_* is the power of transmitting signals, *G_R_* and *G_T_* are the gain of receiving and transmitting antenna respectively, *η_A_* is the efficiency of antenna, *D*(*θ*,*φ*) is the directivity of the receiving antenna, which can be represented as:

(6) $$D(\theta ,\phi )=P_{0}\left[{\left|E_{\theta }(\theta ,\phi )\right|}^{2}+{\left|E_{\phi }(\theta ,\phi )\right|}^{2}\right]\;$$

where $P_{0}=\pi /\int_{0}^{2\pi }\int_{0}^{\pi }\left[{\left|E_{\theta }(\theta ,\phi )\right|}^{2}+{\left|E_{\phi }(\theta ,\phi )\right|}^{2}\right]\sin\theta d\theta d\phi$.

**Figure 2:**
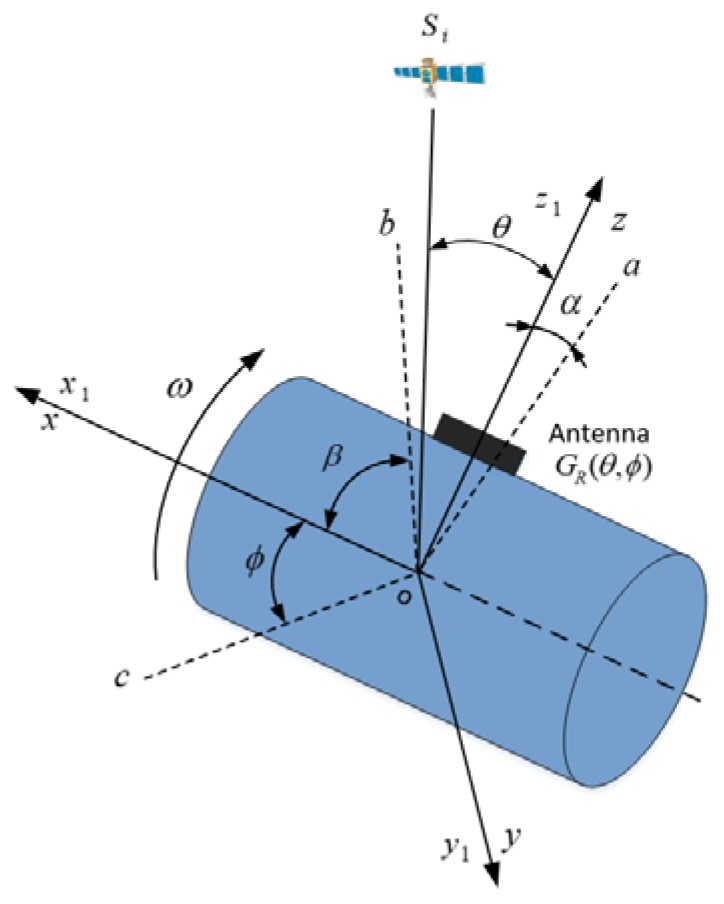
Schematic diagram of the single-patch antenna mounted on a cylindrical vehicle.

Therefore, we can conclude from (4)–(6) that power of the receiver signals received by a single-patch microstrip antenna changes in the form of a sine wave in H surface, and the radiation pattern in Figure 1b also indicates the same results. To thoroughly analyze the relationship between the amplitude of receiver signals and the radiation pattern of the microstrip antenna during rotation, a schematic diagram of the spinning vehicle is presented in Figure 2.

As shown in Figure 2, the antenna, denoted by a black rectangle, is mounted on the surface of a cylindrical vehicle, and the body frame of the vehicle is defined as *ox*_1_*y*_1_*z*_1_, *ox*_1_ along the spinning axis, *oy*_1_ pointing to the phase center of the antenna, and *oz*_1_ completing the right-handed reference frame. The spherical radiation coordinate system of the antenna is defined as *oxyz*, which shares the same origin and axes with body frame *ox*_1_*y*_1_*z*_1_. The only difference between the antenna coordinate system in Figure 1 and Figure 2 is that the origin of the coordinate system in Figure 2 is at the center of the vehicle, not the center of the antenna. However, *θ* in Figure 2 can be approximately equal to the azimuth angle *θ* in Figure 1, because the diameter of the vehicle is short enough to be ignored, as compared to the distance between the vehicle and the satellite, while *φ* in Figure 1 and Figure 2, it is totally the same. So, the radiation pattern of the antenna can still be represented by (1) and (2) with angles (*θ*,*φ*) in Figure 2.

**Figure 3:**
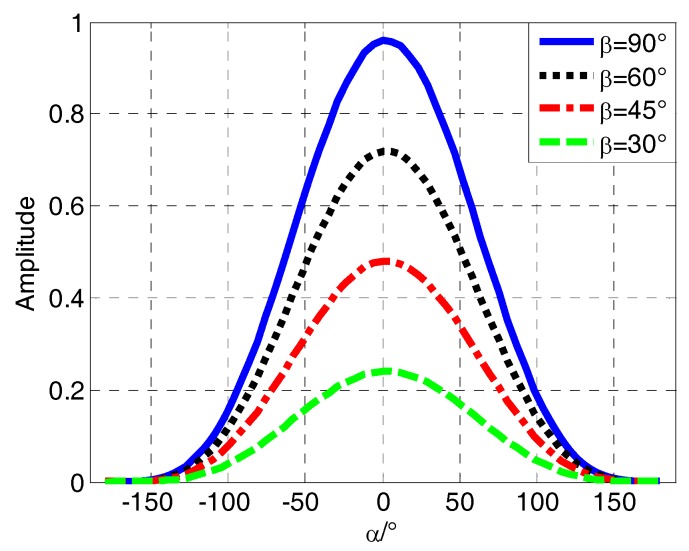
The variation map of signal amplitude received by a rotating antenna.

As we can see from Figure 2, the *i*-th satellite is denoted by *S_i_*. The *oa*, *ob* and *oc* are the projections of the satellite’s line-of-sight (LOS) vector on *y*_1_*oz*_1_, *x*_1_*oz*_1_, and *x*_1_*oy*_1_ surfaces, respectively. *α* is the roll angle in surface *y*_1_*oz*_1_ and *β* is the elevation angle in surface *x*_1_*oz*_1_. When the vehicle rotates in the air, *α* changes periodically from 0 to 360 degrees. To facilitate analysis of the spinning vehicle, (*θ*,*φ*) is replaced by (*α*,*β*). Figure 3 shows the variation of normalized amplitude received by a rotating antenna at different angles of *β*.

So, the power of receiver signals changes in the form of a sine wave, when a vehicle with a single-patch antenna mounted on the side of it, rotates. And the information of the roll angle is also modulated on the variation map of the power of receiver signals, which are called roll modulation signals.

### 2.2. Coordinate Transformations in Roll Angle Measurement

**Figure 4:**
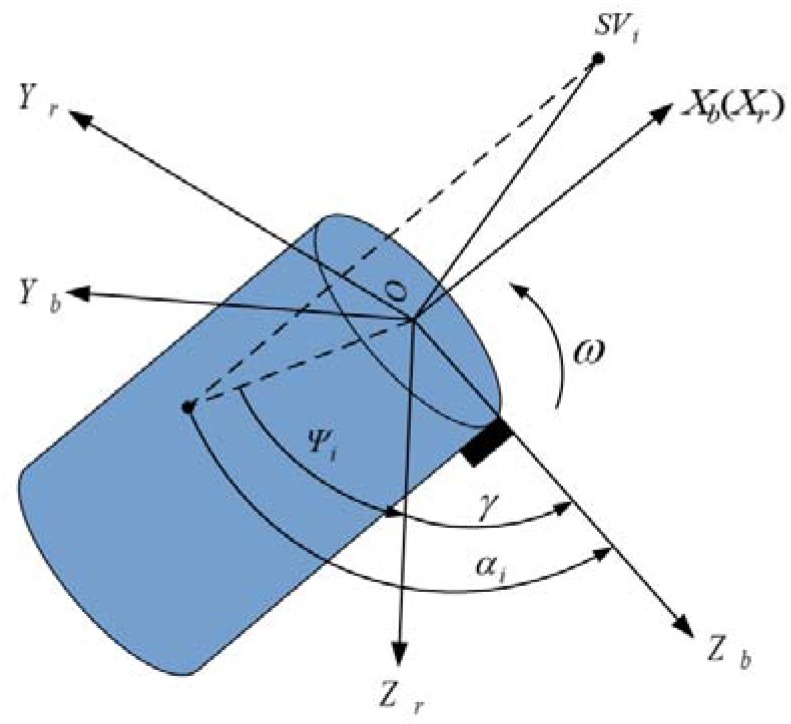
Schematic diagram of roll angle detection.

However, *α* is not the real roll angle that we want. It is related to the true roll angle. To obtain the true roll angle, several coordinate systems are set up on the body of a simplified cylinder vehicle, as shown in Figure 4, in which the single-patch antenna denoted by a black rectangle is mounted on the surface of a cylinder vehicle. *OX_b_Y_b_Z_b_* is a right-handed body coordinate system with origin *O*. The *X*-axis (denoted by *X_b_*) pointing forward, is the spinning axis of the vehicle. *Z*-axis (denoted by *Z_b_*) points to the phase center of the single-patch antenna, and *Y*-axis (denoted by *Y_b_*) is orthogonal to *Z*- and *X*-axes with the usual right-hand rule. *OX_r_Y_r_Z_r_* is a reference coordinate system, and it shares the same origin with the body coordinate system. The *X*-axis (denoted by *X_r_*) points forward and it overlaps with *X_b_*. The *Y*-axis (denoted by *Y_r_*) is parallel to the horizon, and the *Z*-axis (denoted by *Z_r_*) is pointing downward to comply with the right-hand rule.

The body frame spins with the vehicle, while the reference coordinate system is rigidly attached to the spin axis, but not the rest of the vehicle body. The roll angle *γ* is between the *OZ_b_* and *OZ_r_* axis. *α_i_* is the angle between *OZ_b_* axis and the projection of LOS on the *OY_i_Z_i_* surface, which is the phase we are going to obtain from the roll modulation signals.

Therefore, the roll angle *γ* can be described as:

(7) $$\gamma =\alpha_{i}-\psi_{i}\;$$

where *Ψ_i_* can be obtained by the LOS vector in a reference coordinate system, and it differs in circumstances of different satellites and attitudes of the vehicle. The true roll angles detected from different satellites signals should be approximately equal. The roll angles obtained from various satellite signals could be averaged to get a more accurate estimation.

To obtain *Ψ_i_*, the positions of the satellite and vehicle in ECEF (Earth-Centered Earth-Fixed) and geodetic coordinate systems, as well as the attitude of the vehicle, such as yaw and pitch angles, must be known. The definition of ECEF and vehicle-carried NED (North-East-Down) coordinate systems are identical to Cai et al. [21]. *Ψ_i_* is obtained by the following procedures.

1. Get the LOS vector from ECEF positions of the *i*-th satellite and the vehicle, which are obtained by GPS receiver.
  (8) $$P_{LOS}^{i}=P_{s}^{i}-P_{v}={\left[\begin{array}{ccc} x_{s}^{i}-x_{v} & y_{s}^{i}-y_{v} & y_{s}^{i}-y_{v} \end{array}\right]}^{T}={\left[\begin{array}{ccc} x_{e}^{i} & y_{e}^{i} & z_{e}^{i} \end{array}\right]}^{T}\;$$
  where $P_{s}^{i}$ is the *i*-th satellite position, *P_v_* is the vehicle position in the ECEF coordinate system.
2. Transform the LOS vector from the ECEF coordinate system to the vehicle-carried NED coordinate system.
  (9) $$P_{n/e}^{i}=R_{n/e}P_{LOS}^{i}={\left[\begin{array}{ccc} x_{n}^{i} & y_{n}^{i} & z_{n}^{i} \end{array}\right]}^{T}\;$$
  where *R_n/e_* is the rotation matrix from the ECEF frame to the vehicle-carried NED frame, which is given by
  (10) $$R_{n/e}=\left[\begin{array}{ccc} -\sin\varphi_{geo}\cos\lambda_{geo} & -\sin\varphi_{geo}\sin\lambda_{geo} & \cos\varphi_{geo}\\ -\sin\lambda_{geo} & \cos\lambda_{geo} & 0\\ -\cos\varphi_{geo}\cos\lambda_{geo} & -\cos\varphi_{geo}\sin\lambda_{geo} & -\sin\varphi_{geo} \end{array}\right]\;$$
  where *ϕ_geo_* and *λ_geo_* are the geodetic longitude and latitude position of the vehicle respectively.
3. Then transform LOS vector from vehicle-carried NED frame to the reference coordinate system *OX_r_Y_r_Z_r_* which is set up in Figure 4.
  (11) $$P_{r/n}^{i}=R_{r/n}P_{n/e}^{i}={\left[\begin{array}{ccc} x_{r}^{i} & y_{r}^{i} & z_{r}^{i} \end{array}\right]}^{T}\;$$
  where *R_r/n_* is the rotation matrix from the vehicle-carried NED frame to the reference coordinate system, which is given by:
  (12) $$R_{r/n}=\left[\begin{array}{ccc} \cos\psi_{ref} & \sin\psi_{ref} & 0\\ -\sin\psi_{ref} & \cos\psi_{ref} & 0\\ 0 & 0 & 1 \end{array}\right]\left[\begin{array}{ccc} \cos\theta_{ref} & 0 & -\sin\theta_{ref}\\ 0 & 1 & 0\\ \sin\theta_{ref} & 0 & \cos\theta_{ref} \end{array}\right]\;$$
  where *ψ_re_*_f_ and *θ_ref_* are the yaw and pitch angle of the vehicle respectively.
4. Lastly, *Ψ_i_* is obtained by the arctangent value of the LOS vector in the reference coordinate system.
  (13) $$\Psi_{i}=\arctan\left(y_{r}^{i}/z_{r}^{i}\right)\;$$

## 3. Analysis of Roll Modulation Signals

After analyzing the coordinate transformations, we have to construct roll modulation signals, as shown in Figure 3, to get relative roll angle *α*, so that we can obtain the true roll angle from it. In this paper, a magnitude extraction method has been put up, wherein the theoretical derivation and SNR features are also studied.

### 3.1. The Extraction Method of Roll Modulation Signals

**Figure 5:**
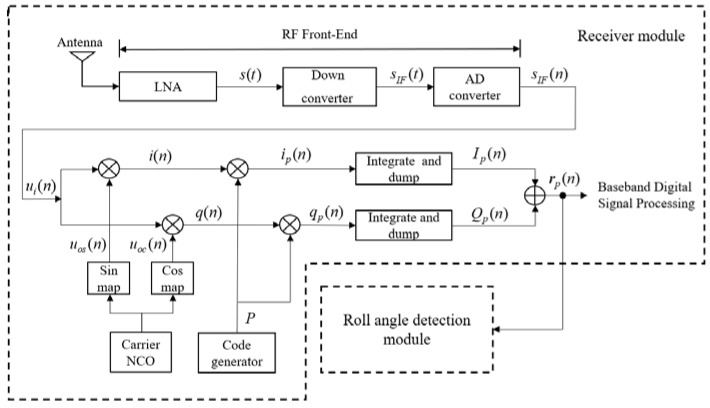
The block diagram of a GPS receiver with a roll angle detection module.

Figure 5 shows the block diagram of a GPS receiver with a roll angle detection module. In a receiver, the power of the *i*-th satellite’s signal is centralized in the correlation results *Ip*(*n*) and *Qp*(*n*) after processed by RF (Radio Frequency) front-end and baseband processing [22]. The correlation results of the *i*-th satellite’s signals can be represented by:

(14) $$I_{p}(n)=a^{\left(i\right)}(\alpha ,\beta )R^{\left(i\right)}(\delta )D^{\left(i\right)}(n)\mathrm{sinc}\left(\frac{1}{2}\omega_{e}T_{carr}\right)\cos\left(\omega_{e}\left(t_{0}+\frac{T_{carr}}{2}\right)+\theta_{e}\right)+n_{I}\;$$

(15) $$Q_{p}(n)=a^{\left(i\right)}(\alpha ,\beta )R^{\left(i\right)}(\delta )D^{\left(i\right)}(n)\mathrm{sinc}\left(\frac{1}{2}\omega_{e}T_{carr}\right)\sin\left(\omega_{e}\left(t_{0}+\frac{T_{carr}}{2}\right)+\theta_{e}\right)+n_{Q}\;$$

where *a*^(*i*)^(*α*,*β*) is the amplitude of receiver signals received by a rotating antenna at the vehicle attitude of (*α*,*β*), *ω_e_* is the angular frequency difference, *θ_e_* is the phase difference between local carrier signal produced by carrier NCO (Numerically Controlled Oscillator) and the input satellite signal, *t*_0_ is the initial time point of the coherent integration, and *T_carr_* is the integration time in the carrier tracking loop of receiver, *n_I_* and *n_Q_* are the noises of each path. *R*^(*i*)^(*δ*) is the self-correlation function of C/A codes. When the code errors do not exist, *R*^(*i*)^(*δ*) reaches the highest value, where *R*^(*i*)^(0) = 1.

The magnitude extraction equation of roll modulation signals can be expressed as follows when the noises *n_I_* and *n_Q_* are ignored:

(16) $$\begin{array}{cc} \left|{\hat{r}}_{p}(n)\right|=\sqrt{I_{p}{\left(n\right)}^{2}+Q_{p}{\left(n\right)}^{2}}= & \left|a^{\left(i\right)}(\alpha ,\beta )R^{\left(i\right)}(\delta )D^{\left(i\right)}(n)\mathrm{sinc}\left(f_{e}T_{carr}\right)e^{j\left[2\pi f_{e}\left(t_{0}+\frac{T_{carr}}{2}\right)+\theta_{e}\right]}\right|\\ & =a^{\left(i\right)}(\alpha ,\beta )R^{\left(i\right)}(\delta )\left|\mathrm{sinc}\left(f_{e}T_{carr}\right)\right|\; \end{array}$$

where *f_e_* = *ω_e_*/2π, sinc(*f_e_T_carr_*) is the correlation loss caused by frequency error, data code *D*(*n*) = ±1 is omitted in the absolute value. Integration time is usually one millisecond in the receiver, *I_p_* and *Q_p_* are also called the one-millisecond accumulation values.

Equation (16) cannot represent the relationship between the roll modulation signals and the relative roll angle *α_i_* directly. To better understand their connections, $\left|{\hat{r}}_{p}(n)\right|$ can be rewritten as:

(17) $$\left|{\hat{r}}_{p}(n)\right|=A_{r}\cos\left(\omega_{r}t+\alpha_{r}\right)+D\;$$

where *ω_r_* = ${\dot{\alpha }}_{i}$, is the angular frequency of antenna rotation; *ω_r_t* + *α_r_* = *α_i_* is the relative roll angle; $A_{r}=\left(a_{\max}^{\left(i\right)}-a_{\min}^{\left(i\right)}\right)R^{\left(i\right)}(\delta )\left|\mathrm{sinc}\left(f_{e}T_{carr}\right)\right|/2$ is the amplitude of the roll modulation signals, and $D=\int_{0}^{2\pi }a^{\left(i\right)}(\alpha ,\beta )R^{\left(i\right)}(\delta )\left|\mathrm{sinc}\left(f_{e}T_{carr}\right)\right|d\alpha /2\pi$ is the direct current (DC) component.

According to (10), it is obvious that the code error *δ* and the frequency error *f_e_* can influence the amplitude of roll modulation signals directly. If the carrier and code tracking loops in GPS receiver are stable during rotation, *R*^(*i*)^(*δ*) and |sinc(*f_e_T_carr_*)| can be considered as constants, and the variation of roll modulation signals only depends on the amplitude *a*^(*i*)^(*α*,*β*), which is directly related to the power of receiver signals.

### 3.2. SNR Analysis of the Roll Modulation Signals

The premise for (16) to be established is that the signal-to-noise ratio (SNR) of one millisecond values have to be high enough. And it is essential for a PLL to track the roll modulation signals with high precision, which requires high SNR as well. Thus, the SNR of roll modulation signals is studied in this section.

**Figure 6:**
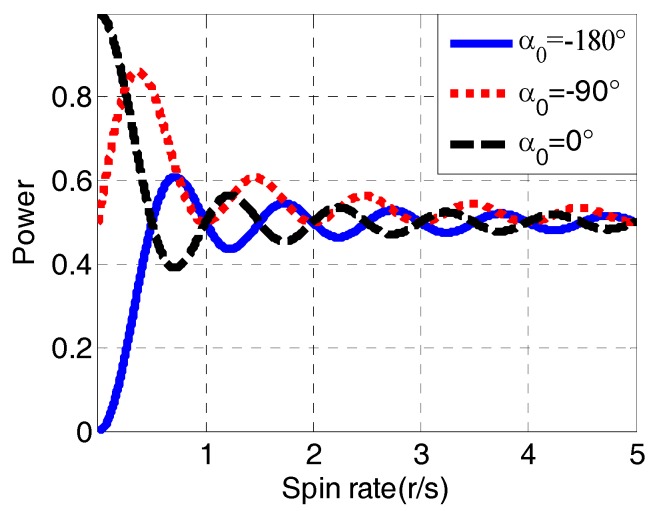
The diagram of normalized average power in one second.

The power of receiver signals can be reduced during antenna rotation and results in attenuation of the power of satellite signals. As shown in Figure 6, it is the normalized average power of received satellite signals in one second at different spin rates with initial relative roll angles *α*_0_.

As we can see from Figure 6, the average power of receiver signals differs when the spin rates and initial angles change. Considering the background of this technology, the spin rate of a spinning vehicle usually exceeds 3 r/s. So, the average power of received satellite signals is approximately equal to the middle value between the maximum and minimum power of the receiver signals. Generally, the maximum and minimum gain of a patch antenna is about 0 dB and −12 dB respectively. While, conversely, the minimum power of received signals is normally 10 times smaller than the maximum power and can be ignored in the average processing. Thus, the average power of receiver signals can be approximately equal to the value of half of the maximum power, which means there is an attenuation of 3 dB in the power compared to the static situation.

**Figure 7:**
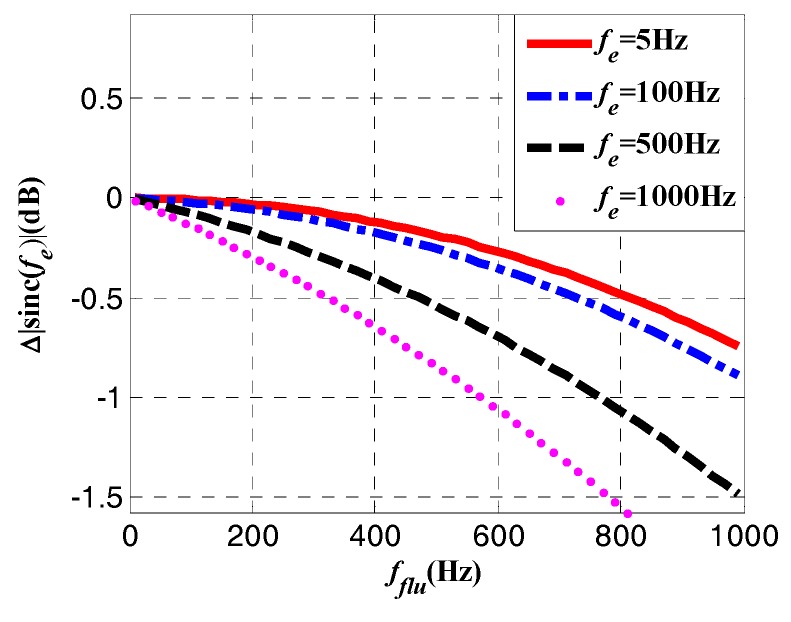
Attenuation caused by fluctuation at different static frequency errors.

In addition to the power attenuation received by the antenna, there is an attenuation in the tracking process. According to (16), the power of roll modulation signals depends on the carrier and code tracking errors at the same time. As for the frequency error *f_e_*, it can only be influenced by the disappearance of satellite signals when the signals are blocked from satellites during rotation, while the code NCO is hardly affected [12]. The block of signals can induce fluctuations on the carrier NCO, which results in the increase of frequency error *f_e_*. The fluctuation induced attenuation can be expressed as:

(18) $$\Delta \left|\mathrm{sinc}(f_{flu})\right|=\left|\mathrm{sinc}(f_{e}T_{carr})\right|-\left|\mathrm{sinc}[(f_{e}+f_{flu})T_{carr}]\right|\;$$

where *f_e_* is the carrier frequency error in static situation, *f_flu_* is the fluctuation induced frequency error during rotation. Figure 7 shows the attenuation caused by fluctuation at different static frequency errors.

**Figure 8:**
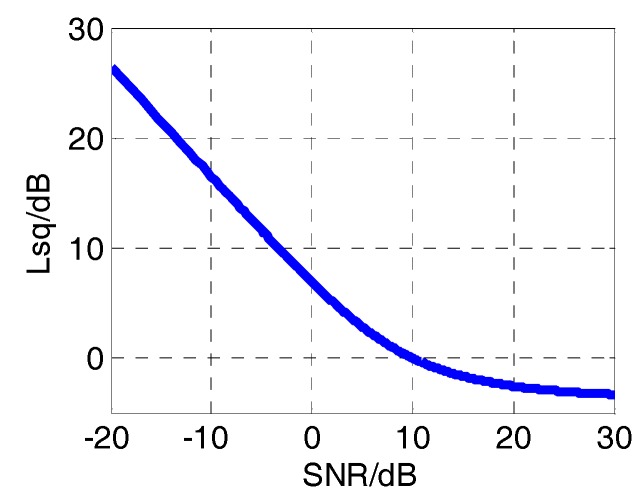
The relationship between squaring loss and the signal-to-noise ratio (SNR) of correlation results.

After the tracking process, there is a square loss *L_SQ_* at the magnitude extraction process, expressed as:

(19) $$L_{SQ}=\mathrm{SN}R_{corr}-\mathrm{SN}R_{roll}\;$$

where SNR*_roll_* is the SNR of roll modulation signals, which can be defined as [23]:

(20) $$\mathrm{SN}R_{roll}={\left[E\left(\sqrt{I_{p}{\left(n\right)}^{2}+Q_{p}{\left(n\right)}^{2}}\right)-E\left(\sqrt{n_{I}{}^{2}+n_{Q}{}^{2}}\right)\right]}^{2}/V\left(\sqrt{n_{I}{}^{2}+n_{Q}{}^{2}}\right)\;$$

where *E*(.) and *V*(.) represent the expectation and variance respectively. SNR*_corr_* is the SNR of correlation results, which is defined as $\mathrm{SN}R_{corr}={\left|{\hat{r}}_{p}(n)\right|}^{2}/\sigma_{n}^{2}$, $\sigma_{n}^{2}$ is the variance of *I_p_*(*n*) or *Q_p_*(*n*). Figure 8 shows the relationship between squaring loss and SNR of correlation results.

As we analyzed above, the power attenuation caused by rotation mainly comes from antenna rotation, frequency error and square loss of magnitude extraction. The overall SNR of roll modulation signals can be represented as:

(21) $$\mathrm{SN}R_{roll}=\mathrm{SN}R_{static}-P_{rotation}-\Delta \left|\mathrm{sinc}(f_{flu})\right|-L_{SQ}\;$$

where SNR*_static_* is the SNR of correlation results in static situation, *P_rotation_* is the power attenuation caused by rotation, equals to 3 dB. Using this formula, we can compute the SNR of roll modulation signals and estimate the tracking errors of PLL, which will be introduced in the following sections.

## 4. Design of Roll Angle Detection Module

Referring to the signal communication processing method, a Frequency-Locked Loop (FLL) assisted Phase-Locked Loop (PLL)-based Costas loop is applied to track the roll modulation signals. The measurements of the tracking loops are the phases and frequencies of roll modulation signals, which correspond to the relative angles and rotational speeds of a spinning vehicle. The theoretical analysis, simulations and error analysis of the loops have also been carried out.

### 4.1. Design of Tracking Loops

**Figure 9:**
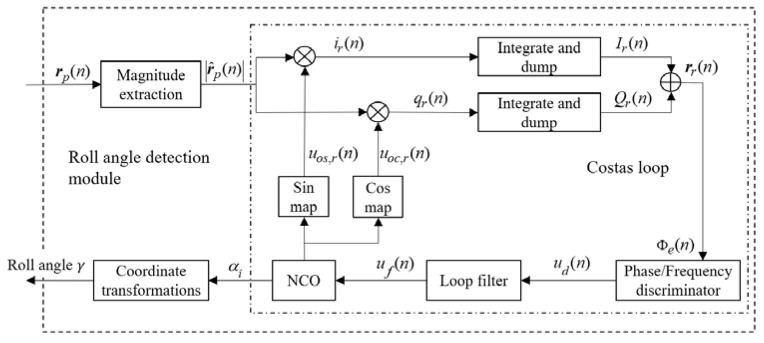
Block diagram of the proposed roll angle detection module.

**Figure 10:**
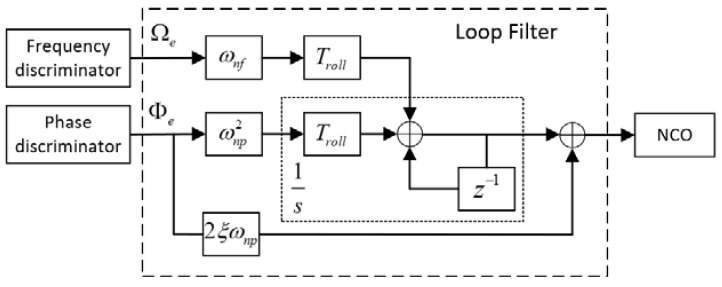
Block diagram of a second-order Phase-Locked Loop (PLL) with first-order Frequency-Locked Loop (FLL) assisted filter.

Figure 9 shows a block diagram of the proposed roll angle detection module. The FLL-assisted PLL based Costas loop contributes the most to the module. FLL-assisted PLL combines the advantages of both tracking loops, which means it can track the phases of input signals precisely under high dynamic stress [24]. A second-order PLL with first-order FLL assisted filter is selected, because both loops are insensitive to constant velocity, while the roll angle measurements here focus on a constant rotational speed with tiny signal dynamic stress to the roll modulation signals. The loop filter is as shown in Figure 10, and there are four parameters that need to be determined. They are damping ratio *ξ*, the natural frequency of PLL *ω_np_*, the natural frequency of FLL *ω_nf_*, and the integration time *T_roll_*. Ω*_e_* is the frequency error, and Φ*_e_* is the phase error between the roll modulation signals and local replicas.

**Figure 11:**
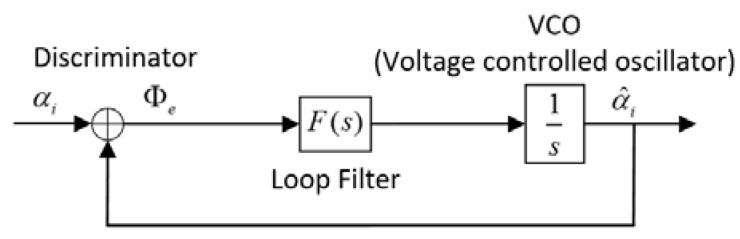
Block diagram of the PLL in S-domain.

In the stable tracking state, PLL plays a more important role than FLL, because it has lower noise bandwidth and makes a more accurate estimate of the phases of input signals. Figure 11 shows the block diagram of a linear PLL in S-domain.

According to Figure 10 and Figure 11, the transfer function of the PLL’s loop filter and the second-order PLL is given by:

(22) $$F_{p}(s)=2\xi \omega_{np}+\frac{\omega_{np}^{2}}{s}\;$$

(23) $$H_{p}(s)=\frac{F_{p}(s)}{s+F_{p}(s)}=\frac{2\xi \omega_{np}s+\omega_{np}^{2}}{s^{2}+2\xi \omega_{np}s+\omega_{np}^{2}}\;$$

respectively.

The noise bandwidth *B*_PLL_ of PLL can be calculated from *ξ* and *ω_np_* by:

(24) $$B_{\mathrm{PLL}}=\int_{0}^{\infty }{\left|H_{p}(j2\pi f)\right|}^{2}df=\frac{\omega_{np}}{2}\left(\xi +\frac{1}{4\xi }\right)\;$$

**Figure 12:**
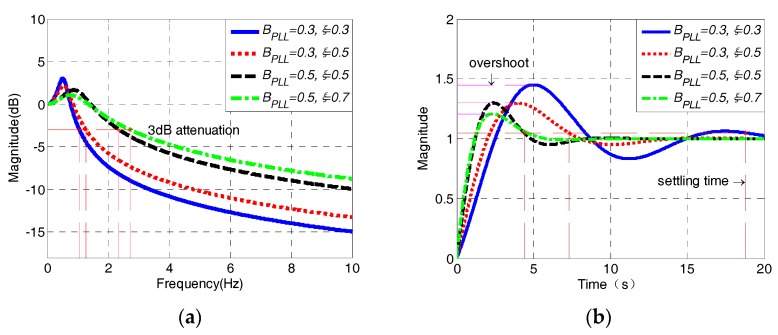
Amplitude–frequency (**a**) and step (**b**) responses of the second-order PLL.

The amplitude–frequency responses and step responses of PLL are simulated with different parameters, which are shown in Figure 12.

As seen from Figure 12a,b, the damping ratio *ξ* and noise bandwidth *B*_PLL_ both determine the cut-off, settling the time and overshoot characteristics of PLL. The larger the damping ratio and the noise bandwidth is, the smaller the settling time and overshoot would be, and the cut-off frequency of PLL could also be increased, leading to a reduction of tracking accuracy. While the frequency of the input signal is between 3 to 300 Hz, the 3 dB attenuation frequency of PLL has to be less than 3 Hz to guarantee the filtering performance when the PLL is used to track the input signals with minimum frequency. In that case, the parameters of PLL must be *B*_PLL_ ≤ 0.5 Hz, *ξ* ≤ 0.7.

The transfer function and noise bandwidth *B*_FLL_ of first-order FLL can be expressed as follows:

(25) $$H_{f}(s)=\frac{F_{f}(s)}{s+F_{f}(s)}=\frac{\omega_{nf}}{s+\omega_{nf}}\;$$

(26) $$B_{\mathrm{FLL}}=\int_{0}^{\infty }{\left|H_{f}(j2\pi f)\right|}^{2}df=\frac{\omega_{nf}}{4}\;$$

**Figure 13:**
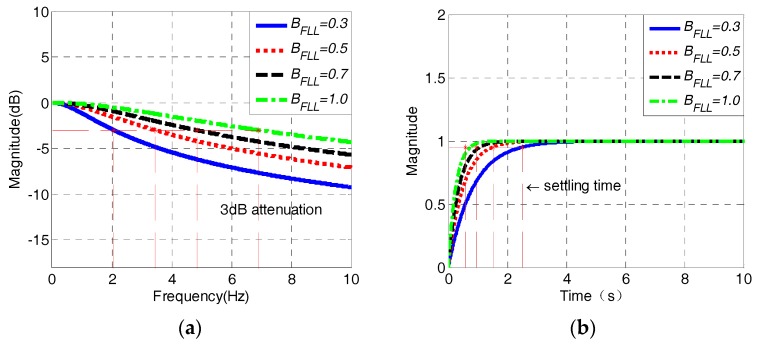
Amplitude–frequency (**a**) and step (**b**) responses of the first-order FLL.

The amplitude–frequency responses and step responses of FLL with different parameters are shown in Figure 13.

As shown in Figure 13a,b, the responses pattern of FLL with different parameters is similar to that of PLL. The larger the bandwidth is, the greater the 3 dB attenuation frequency and the shorter the settling time would be. In order to enable the FLL to track the input signals with all possible frequencies, the frequency at −3 dB point must be less than 3 Hz when it is used to track the input signals with a minimum frequency, where the bandwidth is set to about 0.3 Hz in that case. The function of FLL in the combined tracking loop is to lock the frequency of input signals as quickly as possible, and help pull the loop into the phase-locking state. When the frequency of input signals increases, the bandwidth of FLL can be appropriately enlarged to shorten the settling time and make it faster to track the frequency of input signals.

### 4.2. Parameter Optimization of Tracking Loops

In addition to the noise bandwidth and damping ratio, the integration time *T_roll_* of the integrate and dump process in the roll angle detection module, also has great influence on the tracking loops. As shown in Figure 9, the integrate and dump process works as a low-pass filter to filter out high-frequency noises in *i_r_*(*n*) and *q_r_*(*n*). When the integration time is not large enough, the discriminators could suffer, due to residual errors caused by high-frequency components of *i_r_*(*n*) and *q_r_*(*n*) which eventually lead to a reduction of the tracking accuracy. After the integration process, *I_r_*(*n*) and *Q_r_*(*n*) can be represented as:

(27) $$I_{r}(n)=\sin c(\Omega_{e}T_{roll})\cos\left[\Omega_{e}\left(t(n)+\frac{1}{2}T_{roll}\right)+\Phi_{e}\right]-\sin c(\Omega_{0}T_{roll})\cos\left[\Omega_{0}\left(t(n)+\frac{1}{2}T_{roll}\right)+\Phi \right]\;$$

(28) $$Q_{r}(n)=\sin c(\Omega_{e}T_{roll})\sin\left[\Omega_{e}\left(t(n)+\frac{1}{2}T_{roll}\right)+\Phi_{e}\right]-\sin c(\Omega_{0}T_{roll})\sin\left[\Omega_{0}\left(t(n)+\frac{1}{2}T_{roll}\right)+\Phi \right]\;$$

where Ω*_e_* is the frequency error, Ω_0_ is the sum of frequencies, Φ*_e_* is the phase error and Φ is the sum of phases between the roll modulation signals and local replicas.

Generally, Ω_0_ can be taken as high-frequency components of *I_r_*(*n*) and *Q_r_*(*n*) that can be removed by the integration process, as long as the integration time is more than 5 times the period of input signals. In that case, the longest integration time could reach 1.67 s at 3 Hz, which is barely possible to achieve, because the integration time cannot be too long, due to the limitation of real-time performance and pull-in ranges of the frequency discriminators [22]. The high-frequency components of *I_r_*(*n*) and *Q_r_*(*n*) must be eliminated by other methods, except for extending the integration time. One method to solve this problem is to set the integration time equal to integer multiple of the period of input signals. The formula derivation of this method is given by:

(29) $$\sin c(\Omega_{0}T_{roll})=\sin c(2nf_{r}T)=\sin c(2n)=\sin(2n\pi )/2n\pi =0\;$$

where Ω_0_ = 2*f_r_*, *T_roll_* = *n*/*f_r_* = *nT*, (*n* = 1, 2, …), at the circumstances that the frequency of the input signals *f_r_* has been locked by FLL.

**Figure 14:**
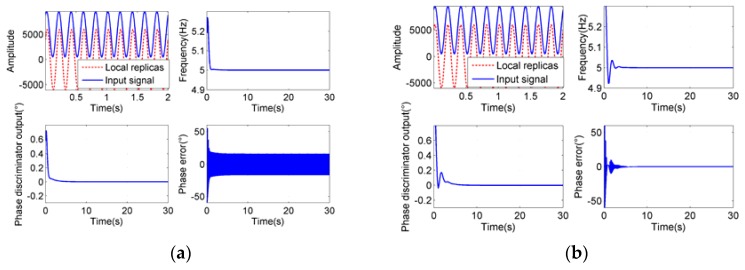
Tracking process at rotational speed of 5 Hz, *B*_FLL_ = 0.3 Hz, *B*_PLL_ = 0.3 Hz, *ξ* = 0.3. (**a**) is the tracking result at *T_roll_* = 100 ms, (**b**) is at *T_roll_* = 200 ms.

**Figure 15:**
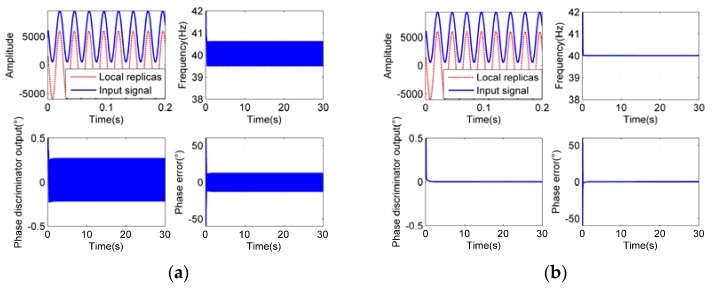
Tracking process at rotational speed of 40 Hz, *B*_FLL_ = 0.3 Hz, *B*_PLL_ = 0.5 Hz, *ξ* = 0.5. (**a**) is the tracking result at *T_roll_* = 13 ms, (**b**) is at *T_roll_* = 25 ms.

**Figure 16:**
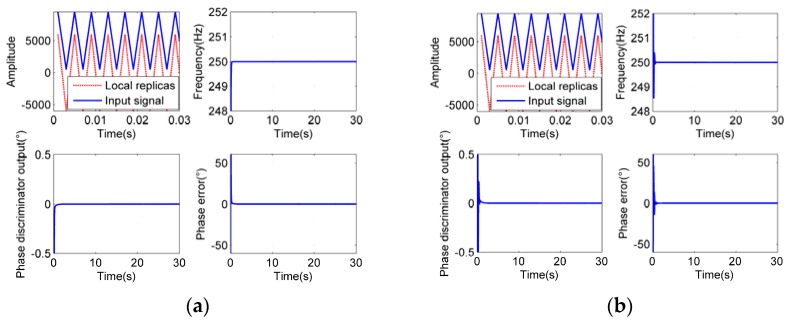
Tracking process at rotational speed of 250 Hz, *B*_FLL_ = 0.5 Hz, *B*_PLL_ = 1.0 Hz, *ξ* = 0.5. (**a**) is the tracking result at *T_roll_* = 20 ms, (**b**) is at *T_roll_* = 50 ms.

To verify the theoretical analysis and figure out the optimal parameter settings, simulations have been carried out. The typical simulation results are shown in Figure 14, Figure 15 and Figure 16. The sampling rate is 1000 Hz.

As we can see from Figure 14, Figure 15 and Figure 16, when the integration time *T_roll_* is half of the period (*T_roll_* = (2*n* − 1) *T*/2, *n* = 1, 2, 3…), the phase errors of tracking results appear to be larger. When the integration time *T_roll_* is integer multiple of the period (*T_roll_* = *nT*, *n* = 1, 2, 3…), the phase errors appear to be the slightest. And when the integration time is more than 5 times larger than the period of input signals (*T_roll_* ≥ 5*T*), it has little effect on the tracking precision, which exactly proves our analysis.

When the frequency of the roll modulation signals changes during the tracking process, and the period of the input signals becomes longer than the original integration time, the tracking accuracy would be worse, or even, the loop could be unlocked. In order to track the frequency-varying signals smoothly and facilitate the parameter settings at different frequencies, a series of fixed parameter settings in different frequency bands are set up. We divide the rotational speeds from 3 r/s to 300 r/s into four groups with division points of 4 r/s, 10 r/s and 40 r/s respectively. The integration times of the first three groups are the maximum periods of the rotational speeds in each group. For they are longer than the other periods in their own group so that the residual errors can be minimized, while the loop is used to track the signals with frequencies of their own group. The integration time of the last group (40 ≤ *f_r_* < 300 r/s) is greater than the longest period of their group, for a fixed integration time of 50 ms is sufficient to meet the acquirements of accuracy and real-time performance at the same time. The optimal choices of other parameters in each group are obtained from extensive simulations with theoretical guidance in the last section. The simulation results in Figure 14, Figure 15 and Figure 16 showed the most appropriate parameter settings that have been discovered, which can satisfy the reasonable settling time and overshoot simultaneously.

The overall optimal parameter settings at different frequencies of FLL-assisted PLL are given in Table 1. The corresponding performance index of the tracking loops is shown in Table 2, where the pull-in ranges of the loops are calculated with respect to a four-quadrant arctangent discriminator. With the presented parameter settings, we can use FLL-assisted PLL to track the roll modulation signals in high precision with reasonable settling time and overshoot. The real-time performance of tracking loops and the flexibility of parameter adjustments can also be ensured at the same time.

### 4.3. Error Analysis of Tracking Loops

For a pure PLL, the dominant sources of phase errors are phase jitter and dynamic stress error. The signal-to-noise ratio at the input of PLL is SNR*_i_*, with the bandwidth of *B_i_*. For there is no data modulation on the roll modulation signals, the square of the RMS phase jitter ${\bar{\sigma }}_{i}{}^{2}$ at the input of PLL can be represented as [25]:

(30) $${\bar{\sigma }}_{i}{}^{2}=1/\left(2SNR_{i}\right)\;$$

The RMS phase jitter ${\bar{\sigma }}_{0}{}^{2}$ at the output of PLL is given by:

(31) $${\bar{\sigma }}_{0}{}^{2}=\left(1/SNR_{i}\right)\cdot \left(B_{\mathrm{PLL}}/B_{i}\right)\;$$

Comparing (30) and (31), the SNR at the output of PLL can be defined as:

(32) $$\mathrm{SN}R_{o}=\mathrm{SN}R_{i}\cdot B_{i}/\left(2B_{\mathrm{PLL}}\right)\;$$

For most cases, the bandwidth of PLL *B*_PLL_ is much narrower than *B_i_*, so we can conclude from (32) that the PLL can improve the SNR at its output. Experimental results have shown that for second-order loops, when SNR*_o_* ≥ 4 (6 dB), a stable operation is generally possible [25]. For example, in this paper, the sampling rate of roll modulation signals is 1000 Hz and the bandwidth of it is approximately *B_i_* = 3 Hz. When *B*_PLL_ is set to 0.5 Hz, the SNR of roll modulation signals must be SNR*_i_* ≥ 1.33 (1.25 dB), so that a stable operation of PLL is possible.

For a second-order PLL, the dynamic stress error can be expressed as [22]:

(33) $$\theta_{d}=1/\omega_{np}^{2}\cdot \left(d^{2}\alpha /dt^{2}\right)\;$$

where *d*^2^*α*/*dt*^2^ is the phase acceleration of the roll modulation signals (deg/s^2^).

So, the overall error of PLL is given by:

(34) $$\sigma_{\mathrm{PLL}}={\bar{\sigma }}_{0}+\theta_{d}\;$$

**Figure 17:**
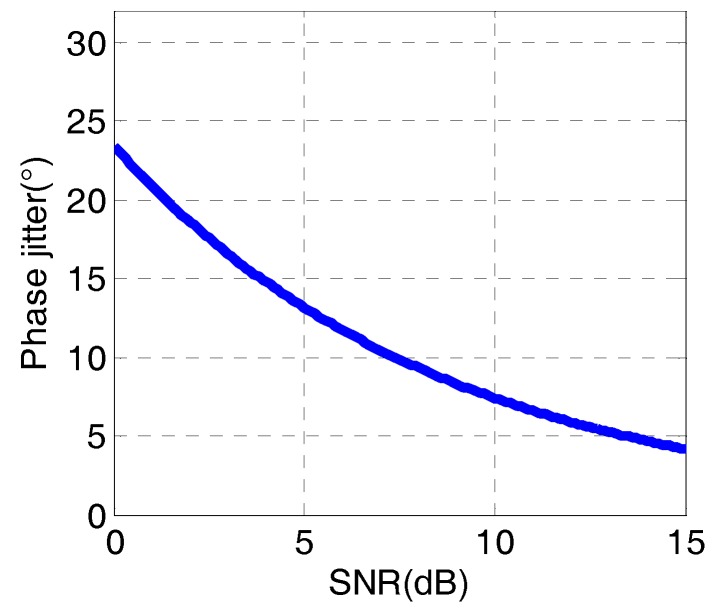
The phase jitter of PLL.

Figure 17 shows the theoretical predictions of the phase jitter at different SNRs of input signals. The bandwidth of input signal is 3 Hz, and the bandwidth of PLL is set to *B*_PLL_ = 0.5 Hz.

As we can see from Figure 17, if the phase jitter of PLL is required to be within 10 degrees, the SNR of the roll modulation signals must be larger than 5.5 (7.4 dB) to ensure the tracking accuracy.

For FLL, the dominant sources of frequency errors show frequency jitter, due to thermal noise and dynamic stress error. The thermal noise of FLL in Hz is given by:

(35) $$\sigma_{t\mathrm{FLL}}=\frac{1}{2\pi T_{roll}}\sqrt{\frac{4FB_{\mathrm{FLL}}}{C/N_{0}}(1+\frac{1}{T_{roll}C/N_{0}})}\;$$

where *F* = 1 at a high carrier-to-noise ratio (*C/N*_0_), *F* = 2 when working near the threshold. The relationship between *C/N_0_* and SNR of input signals is *C/N*_0_ = SNR · *B_i_*.

For a first-order FLL, the dynamic stress error in Hz can be expressed as:

(36) $$v_{d}=1/\left(360\cdot \omega_{nf}^{2})\cdot (d^{2}\alpha /dt^{2}\right)\;$$

According to the rule of thumb for tracking threshold, the 3-sigma jitter of FLL shall not exceed one fourth of the pull-in range of FLL discriminator. Therefore, when the four-quadrant arctangent discriminator is selected, the pull-in range of the discriminator is 1/*T_roll_*, and the tracking threshold of FLL can be described as:

(37) $$3\sigma_{\mathrm{FLL}}=3\sigma_{t\mathrm{FLL}}+v_{d}\leq 1/4T_{roll}\;$$

## 5. Experiment Validation

For the purpose of validating the efficiency and precision of the proposed roll angle detection method, experiments were carried out. An intermediate frequency (IF) signal acquisition system was designed to collect satellite signals received by a single-patch antenna installed on a spinning vehicle. The L1 radio frequency signal is down-converted to the IF signal, sampled and stored in a flash memory unit, which can be processed by an SDR (Software Defined Radio) receiver on PC to obtain the roll modulation signals and test the roll angle detection module. The GNSS SDR software receiver we used was initiated by the Danish GPS Center at Aalborg University and was improved by the GNSS Laboratory at University of Colorado. A Hall Effect sensor was also installed on the spinning vehicle to record the zero-phase point of the roll angle as measurement datum to determine the measurement accuracy of the roll angle measurements. An Analog-to-Digital Converter (ADC) was designed to collect the output of the Hall Effect sensor as well.

### 5.1. Intermediate Frequency Signal Acquisition and ADC Systems

**Figure 18:**
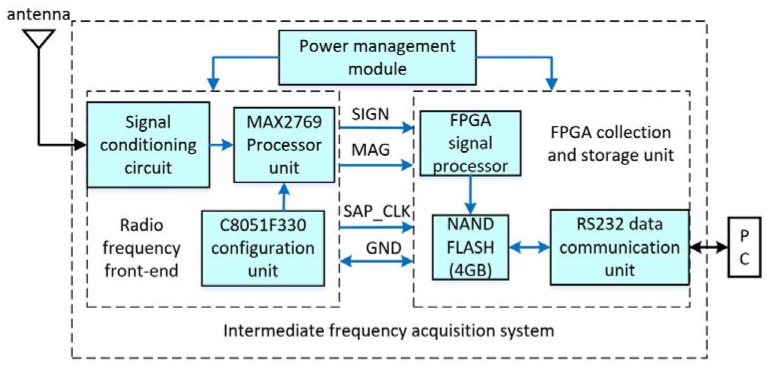
Block diagram of intermediate frequency (IF) acquisition system.

Figure 18 shows a block diagram of the IF acquisition system. The signals from a single-patch antenna were processed in the RF front-end, and then the digital baseband signals were processed in the FPGA (Field-programmable Gate Array) signal processor and saved in the flash memory.

**Figure 19:**
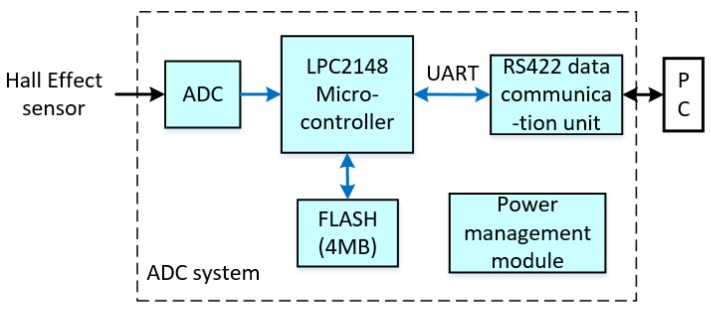
Block diagram of the Analog-to-Digital Converter (ADC) system.

The electrical level of the Hall Effect sensor was recorded by an ADC circuit board with a sampling rate of 100 Hz, which was implemented on the LPC2148 microcontroller with a 10-bit successive approximation analog to digital converter, which is manufactured by NXP Semiconductor (Philips, Eindhoven, Netherlands). The sampling date was stored in a flash memory and read by PC via RS422 after the experiment. The block diagram of the ADC system is shown in Figure 19.

### 5.2. Experiment Description

**Figure 20:**
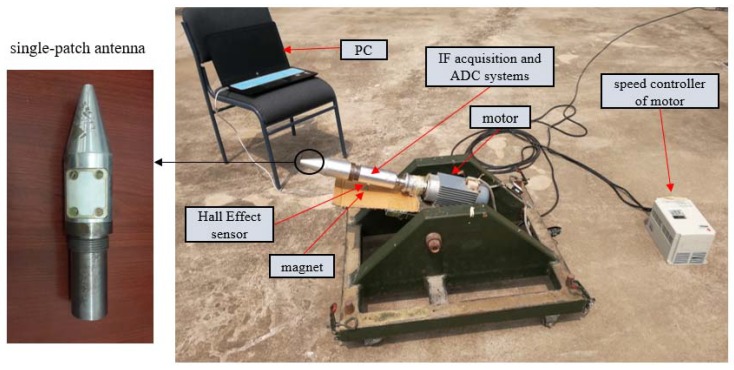
Experiments on a single-axis rotary table.

The installation of the experimental devices is shown in Figure 20. The intermediate frequency acquisition system, ADC system, Hall Effect sensor, and the single patch antenna spun with the vehicle together. When the Hall Effect sensor is activated by the magnet, it produces a low-level pulse as a zero-phase mark point. The magnet is settled below the spinning part of the rotary machine when the center of the antenna is facing the ground, it activates the Hall Effect sensor. The patch antenna was inserted into the vehicle by cutting off the side surface of it, and we adjusted the radiation characteristics of the antenna by integral design with the vehicle, so that the antenna has similar radiation characteristics to the unmounted patch antennas.

The experimental procedure was as follows:

(1) Initialize the Flash of both systems with PC via the serial port. Send signals to start the IF acquisition system (*t*_0_) and the ADC system (*t*_1_) and record their start time;
(2) Ensure the antenna’s boresight is oriented to the zenith and record 90 s of IF data in static situation, record the start time (*t*_2_) and the end time (*t*_3_) as well;
(3) Turn on the rotary machine, the antenna and Hall effect sensor rotates with the vehicle for 120 s with three different speeds listed in Table 3 and record the start time (*t*_3_) and end time (*t*_4_);
(4) Connect the serial port, stop IF signals acquisition system (*t*_5_) and the ADC system (*t*_6_), record the end time of both recording systems, and read the data of them.

### 5.3. Tracking Results

**Figure 21:**
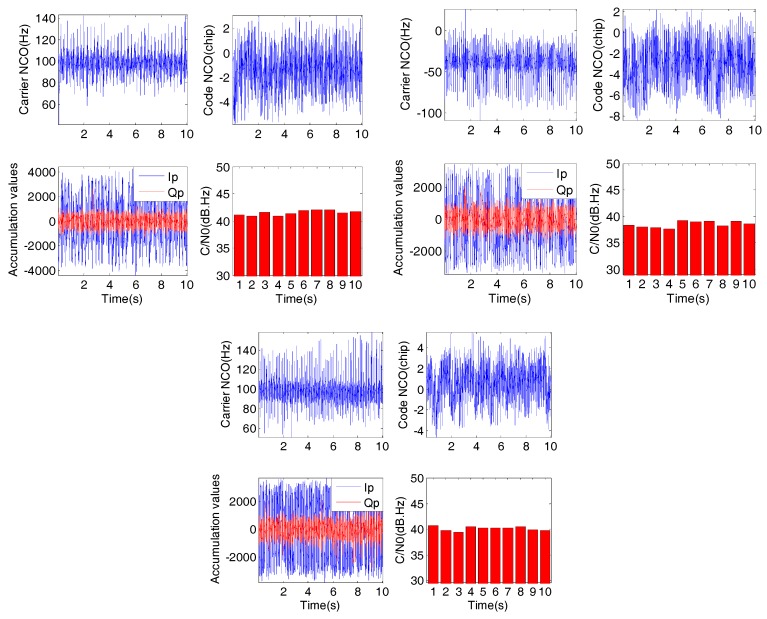
Carrier and code tracking results of different spin rates of 3.8 r/s (top left group), 6.4 r/s (top right group), and 7.5 r/s (bottom group).

Three experiments with different rotational speeds were conducted. The spin rates are 3.8 r/s, 6.4 r/s, and 7.5 r/s respectively. The collected IF signals were processed by two different modules, including the receiver module and the roll angle detection module. The collected signals were processed by an SDR receiver. The carrier tracking loop of the receiver is a second-order FLL-assisted third-order PLL, and the code phase errors were detected by a non-coherent discriminator. The processing results of the carrier and code tracking loops, including carrier NCOs, code NCOs, one-millisecond accumulation values, and the carrier-to-noise ratios (C/N_0_) are shown in Figure 21. As we can see from Figure 21, the carrier-to-noise ratios of the satellite signals are about 40 dB·Hz, which can be considered as strong signals. The carrier and code tracking loops can also work properly during antenna rotation and obtain the one-millisecond accumulation values constantly.

**Figure 22:**
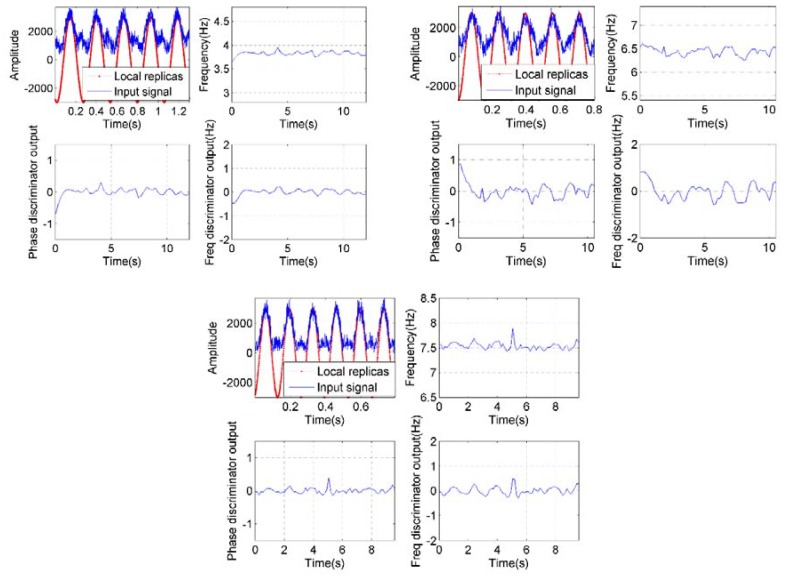
Roll modulation signals tracking results of different spin rates of 3.8 r/s (top left group), 6.4 r/s (top right group), and 7.5 r/s (bottom group).

The one-millisecond accumulation values were then used to construct the roll modulation signals with Equation (16), and they were processed by the roll angle detection module to get roll angle. Figure 22 consists of three groups of panels and shows the tracking results of the roll angle detection module. In each panel group, the top left panel depicts the tracking process of FLL-assisted PLL. The top right panel in each group shows the tracking results of the frequency of the input signal. The phase and frequency discriminators outputs are shown at the bottom of each group. In our experiments, the spin rate was started from 0 to a designated rate and then held. To ensure the tracking accuracy and stability of loops, the signals processed in the roll angle detection module was selected with a constant spin rate.

As shown in Figure 22, the tracking loops are fully capable of tracking the roll modulation signals, despite the roll modulation signals appearing to be a little different from the ideal sine waves. As we analyzed before, the roll modulation signals are directly related to the gain of the antenna. The gain pattern of the antenna we used in the experiments is not exactly the same as the sine wave, so the roll modulation signals seem to be different. To improve the measurement accuracy and tracking stability, the gain pattern of the antenna used to detect roll angle should be designed to match the sine function as much as possible.

### 5.4. Roll Angle Detection Results

**Figure 23:**
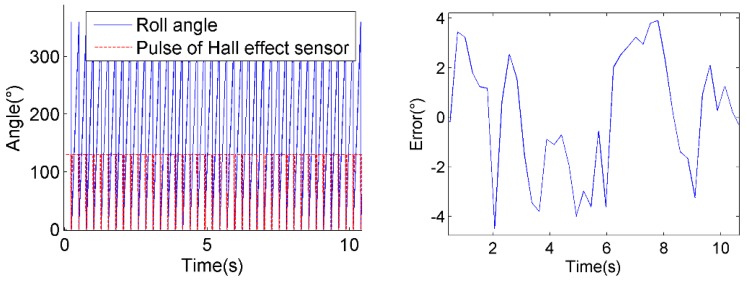

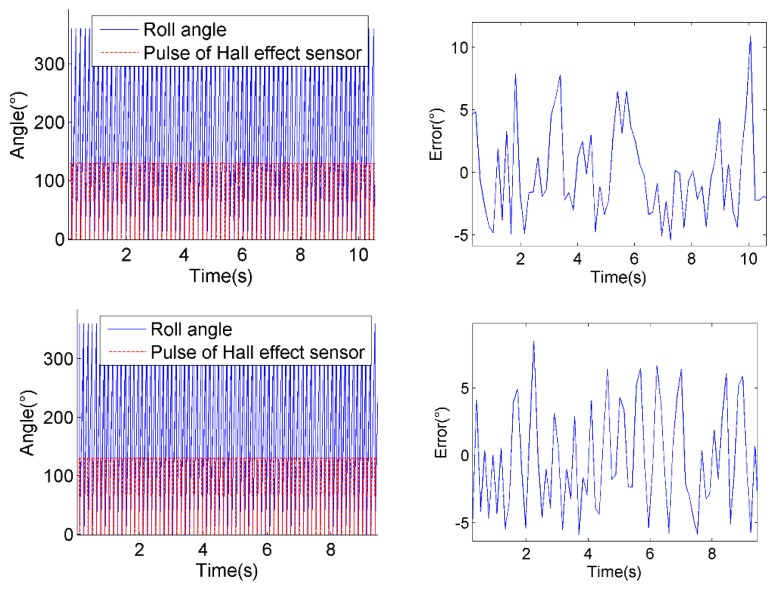
The deviations of estimated roll angles comparing to Hall Effect sensor measurements of 3.8 r/s (top), 6.4 r/s (middle) and 7.5 r/s (bottom).

The detected roll angle is compared to the Hall Effect sensor measurements, as shown in Figure 23, on the left. The deviations of estimated roll angles are shown in Figure 23, on the right. And the overall standard deviations of the roll angle estimation errors are shown in Table 3.

As we can see from Table 3, the average standard deviation of roll angle measurements is 3.3°, which meets the requirements of practical usage. However, it is noticeable that the errors increase as the spin rate rises. It is due to the increase of dynamic stress caused by the instability of motor at high spin rates, which worsens the tracking performance of the second-order phase-locked loops.

## 6. Conclusions

We have achieved roll angle measurement through satellite signals, which are received by a side-mounted single-patch antenna on a spinning vehicle. First, the formation mechanism of roll modulation signals and the coordinate transformations to get real roll angle were analyzed. Then the roll modulation signals were obtained using one-millisecond accumulation values in the GPS receiver as well as analyzing the signal-to-noise ratio. The roll angle detection method based on FLL-assisted PLL was proposed. A first-order FLL assisted second-order PLL was selected and the optimum choices of each parameter were also analyzed. Finally, experiments were conducted to verify the feasibility and accuracy of the roll angle detection method. The experimental results showed that the method can detect the roll angle of a spinning vehicle at a precision of 5 degrees or lower. Further work, including the roll angle measurement experiments at high spin rates and the improvement of tracking accuracy under high dynamic stress, will be investigated.

## Figures and Tables

**Table 1:** Desirable parameters of FLL-assisted PLL.

Rotational Speed/(r/s)	FLL Noise Bandwidth *B*_FLL_/(Hz)	PLL Noise Bandwidth *B*_PLL_/(Hz)	Damping Ratio *ξ*	Integration Time *T_roll_*/(ms)
3 ≤ *f_r_* < 4	0.3	0.3	0.3	333
4 ≤ *f_r_* < 10	0.3	0.3	0.3	250
10 ≤ *f_r_* ≤ 40	0.3	0.5	0.5	100
40 < *f_r_* < 300	0.5	1	0.5	50

**Table 2:** Performance index of FLL-assisted PLL.

Rotational Speed/(r/s)	Settling Time/(s)	3 dB Attenuation Frequency/(Hz)	Overshoot	Pull-In Range/(Hz)
3 ≤ *f_r_* < 4	18.76	1.04	1.45	±1.5
4 ≤ *f_r_* < 10	18.76	1.04	1.45	±2
10 ≤ *f_r_* ≤ 40	4.38	2.35	1.30	±5
40 < *f_r_* < 300	2.3	4.70	1.21	±10

**Table 3:** Standard deviations of roll angle measurements.

Group	Nominal Rotational Speed/(r/s)	Real Rotational Speed /(r/s)	Standard Deviation/(°)
1	5	3.8	2.5
2	8	6.4	3.7
3	10	7.4	4.2
Average deviation			3.3
